# Recognition of Gait Alterations Induced by Alcohol-Impairment Simulation Goggles Using Smartphone Accelerometer Signals

**DOI:** 10.3390/s26103038

**Published:** 2026-05-12

**Authors:** Paweł Marciniak, Mariusz Zubert

**Affiliations:** Department of Microelectronics and Computer Science, Lodz University of Technology, 93-005 Lodz, Poland; mariusz.zubert@p.lodz.pl

**Keywords:** wearable inertial sensors, smartphone sensing, gait classification, drunk goggles, alcohol goggles, FVG, LSTM, CNN, self-attention

## Abstract

**Highlights:**

**What are the main findings?**

- Neural network models applied to smartphone accelerometer data can distinguish gait patterns associated with simulated visual impairment.
- Outdoor data acquisition in an open environment produced more discriminative gait data than the initial structured indoor setup.

**What are the implications of the main findings?**

- Smartphones may serve as low-cost, widely available platforms for exploratory, low-stakes screening or educational applications related to gait impairment.
- The released dataset can support future work on benchmarking of inertial-sensor-based models (using accelerometer and gyroscope data) and more rigorous subject-wise validation.

**Abstract:**

The reliable identification of impairment relevant to safety-critical activities remains a significant challenge for public safety, motivating the exploration of unobtrusive and widely accessible sensing technologies. This study examines the viability of utilising inertial data acquired from consumer-grade smartphones to characterise gait disturbances associated with simulated visual impairment. The study simulates intoxication-related effects using alcohol-impairment goggles and does not involve the measurement of real alcohol intoxication. Two supervised experimental protocols were conducted in which participants traversed predefined walking routes under normal conditions and while wearing alcohol-impairment simulation goggles representing five manufacturer-declared blood alcohol concentration (BAC)-related goggle conditions plus a no-goggles control condition. An initial indoor trial, conducted in a structured corridor environment, yielded limited discrimination of gait dynamics due to strong spatial and visual stabilisation cues. To address this limitation, a subsequent outdoor experiment was conducted along a 100 m path lacking prominent visual reference points, resulting in motion patterns that more closely reflect unconstrained, real-world locomotion. Tri-axial accelerometer and gyroscope signals were recorded using smartphones, followed by artefact removal, segmentation, and standardisation to ensure inter-trial comparability. The resulting curated dataset comprises 290,919 multi-channel samples derived from 96 walking trials involving 16 participants and is released as an openly accessible resource to support further research in gait analysis and classification of gait alterations associated with simulated impairment. Model evaluation was performed using an 80/20 train–test split conducted within each traversal, with training and test windows originating from the same participant and walking session. Consequently, the reported results reflect within-subject performance instead of subject-independent generalisation. Multiple deep learning architectures combining convolutional feature extraction, bidirectional long short-term memory layers, and self-attention mechanisms were systematically evaluated. Using a subject-dependent evaluation protocol, the best-performing architecture achieved an accuracy of 71.4% and a weighted F1-score of 71.5% in distinguishing gait patterns associated with different levels of simulated visual impairment. The best-performing architectures yielded classification performance consistent with exploratory, low-stakes assessment of gait alterations associated with simulated visual impairment, using accelerometer data alone. These findings illustrate the feasibility of using smartphones as auxiliary tools for exploratory, low-stakes screening or educational applications and contribute a publicly released dataset and benchmark results to facilitate methodological advancement in inertial sensor-based gait impairment analysis.

## 1. Introduction

Alcohol consumption remains a prevalent phenomenon in both Europe and the United States. According to the World Health Organisation (WHO), adults in the WHO European Region consume, on average, 9.2 litres of pure alcohol per year, making it the region with the highest level of alcohol consumption worldwide [1]. Alcohol consumption also remains high in the United States, where the corresponding figure is 8.7 litres [2]. Notwithstanding sustained prevention efforts, alcohol use continues to impose a substantial public health burden, including important implications for road safety.

In the operation of non-commercial motor vehicles, alcohol intake prior to driving exerts a direct and substantial detrimental effect on performance and constitutes one of the principal threats to traffic safety. Alcohol acts as a depressant on the central nervous system, causing delayed reaction times, impaired motor coordination, restricted visual fields, and reduced accuracy in judging distance and speed. Even a relatively low blood alcohol concentration (BAC) can alter human perception and behaviour, markedly compromising a driver’s situational awareness and responsiveness. Reported estimates indicate that, at a BAC of 0.02%, the risk of involvement in a crash may approximately double, whereas at 0.05% it may increase several-fold [3]. Representative, broadly estimated values, calculated according to the formula of Erik M. P. Widmark [4,5] and based on data in [6], are provided in Table 1. Comparable compilations can be found in ref. [6], which includes an analysis of BAC effects on driver reaction time, and in refs. [7,8], which present detailed investigations of BAC impacts on vehicle operation.

According to WHO data, drink-driving is a major contributor to road trauma in Europe [9]. In Poland, the 2023 National Police Headquarters report indicates that intoxicated drivers were responsible for more than 1500 accidents, causing 207 fatalities and more than 1800 injuries [10]. Many drivers remain unaware that alcohol may remain in the bloodstream for several hours, potentially persisting until the following morning after evening consumption.

Effective countermeasures against drink-driving require not only rigorous law enforcement but also broad social education and the promotion of responsible attitudes. Only complete sobriety at the wheel can ensure the safety of both the driver and other road users.

The regulation of alcohol-related restrictions is not confined to driving; it extends to other professions where there exists a tangible risk of loss of life, severe injury to others, or substantial social and economic repercussions. In the United States, relevant regulations apply, for example, to:

- Aircraft piloting, air traffic control, and federal aviation personnel (legal basis: 14 CFR §91.17; 14 CFR §120.37; and 14 CFR Part 120)
- Professional commercial motor vehicle drivers (legal basis: 49 CFR 382.201; 49 CFR 392.5; and FMCSA guidelines)
- Federal railroad machine operations (legal basis: 49 CFR 219.101)
- Pipeline and hazardous materials operations (legal basis: 49 CFR 199.219; 199 Subpart C; Part 199; Part 40)
- Public transport operations under federal regulation (legal basis: 49 CFR 655, Subpart E, and FTA regulations)
- Maritime navigation and seafaring (legal basis: 33 CFR 95.020/Part 95)

Permissible BAC thresholds for non-professional drivers vary considerably across jurisdictions, reflecting differences in national regulatory frameworks. In Poland, the legal limit for drivers is BAC < 0.02%. In Germany and France, the threshold is BAC < 0.05%, whereas in England, Wales, and Northern Ireland, the corresponding limit for non-professional drivers is BAC < 0.08%. Concerning maritime navigation in the United States, the permissible BAC threshold is BAC < 0.08% for recreational operators, while a stricter limit of BAC < 0.04% applies to commercial vessel operators. These thresholds are not solely legal constructs; they are also associated with measurable impairments in reaction time and with the predicted incidence of hazardous events, particularly in the domain of road traffic safety. These BAC levels may also be associated with changes in reaction times and the projected frequency of hazardous incidents on the road [7,8,11].

Against this background, the present study seeks to address the problem of sensory-induced gait alterations relevant to safety-critical activities by investigating whether machine learning methods can detect and classify gait changes induced by visual impairment simulated with commercially available alcohol-impairment goggles, using data acquired from inertial sensors embedded in smartphones. Importantly, the study does not aim to detect actual alcohol intoxication, but rather to analyse gait adaptations arising from controlled, vision-based impairment that has been widely employed in educational and experimental settings. This distinction is important because previous research may suggest that alcohol-related impairment states can be inferred from smartphone accelerometer signals using deep learning models trained against transdermal alcohol concentration measurements, thus providing preliminary support for the broader feasibility of mobile inertial sensing in impairment-related classification tasks [12]. For this purpose, we draw upon previous research on gait alterations induced by alcohol-impairment goggles presented in ref. [13]. During the experimental phase, a dataset of human gait measurements was collected using mobile-phone sensors as participants walked both without goggles and while wearing alcohol-impairment simulation goggles corresponding to six manufacturer-declared BAC-related conditions; the relationship between these simulated conditions and BAC levels reported in the literature is discussed in a subsequent section. The approach presented in this study constitutes an initial demonstration of the problem, indicates a possible direction for addressing it, and makes the collected experimental data available to other researchers. A mobile application implementing such algorithms could be conceptually explored as an auxiliary, low-stakes screening or educational instrument, instead of a substitute for certified measurement devices, such as police breathalysers. Its principal advantages include accessibility and convenience, since a mobile phone is almost always available and may enable rapid self-assessment of sobriety prior to driving; educational and preventive value, because even an imperfect warning indicating elevated impairment could discourage a user from driving; and the possibility of integration with other applications designed to remind the user to rest after alcohol consumption, issue driving warnings, or activate engine interlock systems in suitably equipped vehicles. Various neural network architectures are investigated, and their performance is systematically compared in this study.

Importantly, the present study does not measure real alcohol intoxication or blood alcohol concentration. Instead, gait alterations are induced using alcohol-impairment simulation goggles, and the observed effects should be interpreted as adaptations to simulated visual impairment instead of physiological effects of alcohol consumption.

Recent advances in wearable gait analysis have established inertial sensing as a practical bridge between laboratory-grade motion analysis and real-world mobility assessment. Contemporary studies show that wearable IMUs (Inertial Measurement Units, e.g., containing an accelerometer, a gyroscope, and a magnetometer) and smartphone-embedded accelerometers can quantify spatiotemporal gait parameters with clinically meaningful accuracy and enable robust walking recognition in ecologically valid settings. At the same time, the literature has expanded beyond classical parameter extraction toward gait classification, gait phase/event detection, and gait-based biometric recognition using deep neural networks. In particular, convolutional, recurrent, and hybrid architectures have been shown to learn discriminative representations directly from raw inertial signals, thereby improving performance under realistic variability in device placement, walking speed, and unconstrained acquisition conditions. This development is important for the present study because it demonstrates that commodity mobile sensors are no longer used solely for generic activity monitoring, but are increasingly being applied to gait-specific inference tasks that require sensitivity to subtle temporal and kinematic changes [14,15,16,17,18,19,20].

However, despite this progress, most previous wearable studies have focused either on clinical or pathological gait assessment or on identity-oriented gait recognition and authentication [14,15,16,17,18,19,20]. Considerably less attention has been paid to datasets specifically designed to capture gait alterations induced by controlled visual sensory perturbations in otherwise healthy individuals. The present study addresses this gap by introducing a smartphone-based dataset and a classification framework focused on gait changes associated with simulated visual impairment, thereby complementing both the clinical wearable gait literature and the gait biometrics literature with a sensory-perturbation perspective; the relationship between the simulated alcohol-impairment conditions and BAC-corresponding levels, as reported in the literature, is discussed in a subsequent section.

## 2. State-of-the-Art Classification Methods in Wearable Gait Analysis

Wearable and smartphone-embedded inertial sensors are now widely used in gait analysis, as they enable low-cost, mobile, and repeatable acquisition of locomotion signals outside specialised laboratory environments [14,15,21]. Recent studies indicate that wearable IMUs and smartphone-based accelerometers can provide clinically and methodologically meaningful gait information, including walking recognition, gait-event detection, and estimation of spatiotemporal gait parameters, while preserving ecological validity under real-world acquisition conditions [14,15,17,19,22,23,24]. This development is particularly important for the present study, as it demonstrates that commodity mobile devices are no longer used solely for generic human activity recognition but are increasingly used for gait-specific inference tasks that require sensitivity to subtle temporal and kinematic variations. A summary of the most relevant studies is presented in Table 2.

From a sensor configuration perspective, recent literature indicates a shift from highly instrumented laboratory setups to more transferable, consumer-grade sensing solutions [14,15,21,23]. Although multi-IMU systems still provide detailed kinematic descriptions, smartphone-based and single- or few-sensor wearable approaches are increasingly preferred when the objective is robust mobile gait assessment under naturalistic conditions [14,19]. This issue is directly relevant to the present study, which focuses on smartphone accelerometer data instead of on specialised motion-capture systems or complex multi-sensor laboratory configurations.

A particularly relevant branch of the literature concerns gait recognition and gait-based authentication using wearable inertial data [16,18]. Within this line of research, accelerometer and gyroscope signals are treated not only as sources of biomechanical descriptors, but also as behavioural biometric signatures that can support subject verification, identification, and continuous authentication [16,18]. Recent studies have shown that gait-based authentication using smartphone sensors can achieve high performance even with short acquisition windows and variable carrying positions, confirming that mobile gait signals contain stable and discriminative information suitable for classification tasks [16].

Within the gait-recognition literature, deep learning has emerged as one of the dominant modelling paradigms [15,16,17,19,20,21,25]. Convolutional neural networks are widely used to learn local discriminative patterns from inertial sequences, recurrent architectures such as LSTMs are employed to model temporal dependencies, and hybrid approaches combine both advantages when capturing both cyclic gait structure and longer-range temporal information jointly [16,19,20]. This is consistent with broader trends in wearable gait modelling, in which deep architectures increasingly outperform conventional pipelines based on hand-crafted features, particularly in settings affected by sensor-placement variability, environmental noise, or inter-subject differences [16,17,19].

Recent smartphone-based studies further indicate that deep learning is no longer confined to identity-related gait recognition, but also supports fine-grained gait-analysis tasks such as gait-event detection and the estimation of spatiotemporal parameters [17,19,26]. This is important for the present study because it provides methodological justification for evaluating neural architectures on mobile inertial gait data, even when the target classes are not user identities or pathological diagnoses, but experimentally induced gait alterations.

**Table 2:** Selected recent studies on wearable gait analysis, gait recognition, and deep learning methods most relevant to the present work.

Reference	Platform/ Sensor	Task	Model/ Approach	Main Contribution	Relevance to the Present Study
Kotas 2019 [27]	Tensometers and inertial sensors	Agreement between posturography methods	Method-comparison/agreement study	Evaluates agreement between conventional teso-metric posturography and inertial-sensor-based posturography.	Provides methodological support for the validity of inertial sensors in balance-related assessment, though it is less directly related to gait classification than the other cited works.
Di Biase 2020 [28] Zhang 2024 [17]	Wearable sensors/IMUs	Quantitative assessment of Parkinsonian gait	Review of diagnostic markers, ML-based clinical gait scoring, and wearable quantitative gait analysis	Together, these works show that wearable gait markers can support diagnosis, subtype differentiation, disease-severity monitoring, and automated clinical assessment in Parkinson’s disease.	Relevant as clinically oriented evidence that wearable sensing captures subtle but meaningful gait alterations, supporting the broader rationale for classification of experimentally altered gait in the present study.
Shahar 2021 [22] Lee 2025 [19] Prisco 2025 [24] Gil-Martín 2020 [29]	Single smartphone sensor or single wearable IMU, including lumbar-mounted IMUs	Gait-event detection and spatiotemporal gait-parameter estimation	Agreement/validation study plus hybrid threshold-based/deep learning, CNN-RNN gait-event models, and a single-lumbar-IMU algorithm	These studies show that accessible single-device setups can estimate clinically relevant gait parameters and detect gait events with good accuracy, including pocket-carried smartphones, waist-worn sensors, and lumbar-mounted IMUs.	Directly supports the present study’s choice of compact inertial setups and motivates the use of single-device gait-specific modelling for spatiotemporal analysis beyond generic activity recognition.
Prasanth 2021 [21] Prisco 2024 [14]	Wearable IMUs/inertial sensors	Methodological review of gait analysis and real-time gait detection	Systematic and narrative reviews of validation, sensor placement, and gait-event pipelines	Collectively show the methodological maturity of inertial sensing for gait analysis, highlighting validation against reference systems, the importance of sensor placement, and the central role of IMUs in real-time gait-event detection.	Provides consolidated methodological background for the present study by supporting inertial sensing as a valid platform for mobile gait analysis and by positioning smartphone-based gait-event modelling within broader wearable-sensing pipelines.
Kamiński 2022 [30] Kamiński 2025 [31]	IMU/six MediPost inertial sensors (with single-sensor and six-sensor variants)	Detection of balance disorders related to vestibular impairment	Artificial neural network, comparison with analytical single-sensor and multi-sensor approaches	Showed that ANN-based inertial-sensor approaches can detect subtle movement abnormalities associated with vestibular dysfunction, with better diagnostic performance for the multi-sensor configuration, while simpler one-sensor variants may still be useful for screening.	Relevant as evidence that inertial-sensor data combined with neural models can detect subtle movement alterations related to sensory dysfunction, which supports the broader rationale for classifying experimentally induced gait/balance changes in the present study.
Gil-Martín 2022 [12]	Smartphone-embedded sensors, with transdermal alcohol concentration (TAC) measured using an ankle-worn alcohol sensor.	Binary classification of intoxication status and estimation of transdermal alcohol concentration (TAC) based on accelerometer measurements.	CNN-based model trained on FFT-derived accelerometer windows for intoxication classification and TAC estimation.	Proposed a robust smartphone-based motion biomarker and improved performance on the Bar Crawl dataset.	It supports the feasibility of smartphone-based inertial sensing for impairment-related classification, although it concerns actual intoxication instead of simulated sensory impairment.
Choi 2023 [16]	Smartphones, smartwatches, smartphone IMUs	Gait biometrics, authentication, and person identification	CNN-based authentication, smartwatch post-processing, and hip-angle-based ML identification	These studies show that consumer-device gait signals contain discriminative user-specific information and that performance can be improved through deep learning, post-processing, or biomechanical feature engineering.	Supports the claim that mobile inertial gait signals are sufficiently distinctive for classification, reinforcing the broader feasibility of learning meaningful gait signatures from smartphone data.
Li 2023 [25] Hwang 2024 [32] Palazzo 2025 [20]	Body-worn or multi-sensor IMU systems	Pathological/abnormal gait classification	Deep-learning and ML classifiers (CNN-BiLSTM, SVM/RF/XGB, and related deep-learning frameworks)	These studies demonstrate that inertial data can distinguish abnormal gait patterns arising from neurological or joint impairments, and that sensor fusion or targeted feature selection can improve classification performance.	Provides strong methodological support for using IMU signals to classify altered gait patterns, which is directly aligned with the present study’s classification objective.
Straczkiewicz 2023 [15] Kim 2025 [23]	Smartphones, smartwatches, wearable accelerometers, smartphone IMU	Walking recognition and walking-related behavioural-state detection	Signal-based walking recognition and personalised behaviour modelling	Together, they demonstrate robust recognition of walking-related states across heterogeneous consumer devices, body locations, and realistic usage conditions, including handheld and pocketed smartphone use.	Strengthens the ecological validity of the present study by showing that commodity mobile devices can support gait-related inference outside tightly controlled laboratory settings.
De Marsico 2024 [18]	Wearable accelerometer signals	Gait biometrics/recognition	Feature-based gait biometric modelling	It showed that wearable gait-recognition performance depends strongly on the training setup and session design, not only on the classifier itself.	Supports the importance of experimental protocol and dataset design in gait-related classification tasks.

Consequently, the most relevant context for the present work is not the broad field of wearable sensing applications, but the more specific intersection of smartphone-based gait analysis, gait recognition, and deep-learning-based classification of inertial signals [12,14,15,16,17,18,19,20]. Existing research has focused primarily on clinical or pathological gait assessment [27,30,31], generic walking recognition, or biometric user authentication [14,15,16,17,18,19,20]. In contrast, the present study examines gait alterations induced by controlled visual impairment and provides a dataset specifically designed to analyse the effects of sensory perturbation in smartphone accelerometer recordings. In this sense, the contribution of the present work is to extend the current wearable gait literature by introducing a classification setting in which class separation is driven by experimentally manipulated perceptual constraints instead of by subject identity, routine activity type, or disease status [29,32].

## 3. Design of the Research Experiment and the Initial Experimental Study

**Figure 1:**
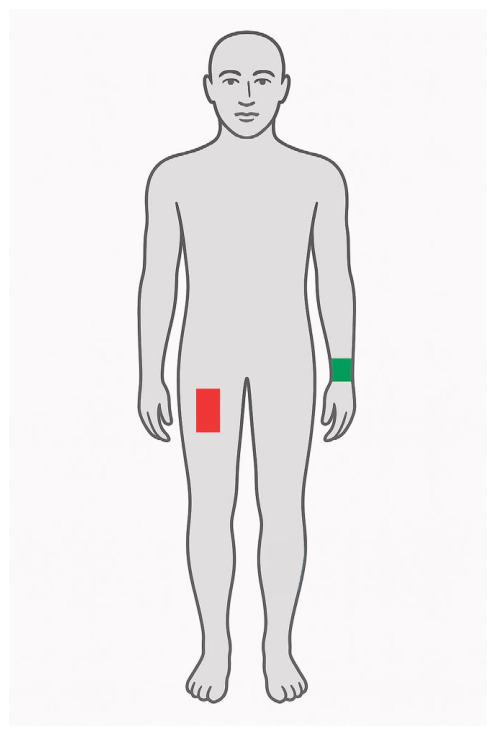
Visualisation of the locations of mobile phones recording movement via sensors integrated into the devices. The red box denotes the position of a Samsung Galaxy S23 placed in the right trousers pocket, while the green box denotes the position of another Samsung Galaxy S9.

The central focus of the presented study is the relationship between human gait, as quantified by smartphone sensors while participants walked along a controlled, predefined schema, and the degree of sensory visual distortion induced by the Drunk Busters Totally Wasted Goggles (commonly termed alcohol or beer goggles; Drunk Busters of America, LLC, Brownsville, TX, USA; see Table 2). A comparable device, Fatal Vision^®^ Alcohol Goggles (FVGs; Innocorp, Ltd, Verona, WI, USA), exerts a similar effect on visual perception and has been used to replicate alcohol-related driving impairment in populations for whom actual alcohol administration would be unethical (e.g., pre-licensed drivers). The proposed experiment was conducted using all available smartphone sensors; however, in this study, only accelerometer data are utilised for model development. As part of the research undertaken, two experiments were designed in which participants traversed a straight segment of a pathway, came to a stop, executed a turn, and returned along the same route to the starting point. Each trial was recorded using two mobile devices (see Figure 1):

- A Samsung Galaxy S23 placed in the right trousers pocket (red rectangle in Figure 1);
- A Samsung Galaxy S9 affixed to the left wrist (green rectangle in Figure 1).

The study employed five different models of Drunk Busters alcohol-impairment simulation goggles, each designed to simulate a specific degree of intoxication, as indicated in Table 3.

Although two smartphones and multiple sensor modalities were used during data acquisition, the analyses presented in this paper utilise data from a single smartphone placed in the right trousers pocket.

In the initial iteration of the experiment, the study was conducted indoors, specifically within a narrow corridor measuring 280 cm in width. Data were acquired during an 18 m linear walk, followed by a return along the same path. A custom-developed Android mobile application was used to record data from the devices’ built-in sensors (accelerometer and gyroscope) at a sampling frequency of 50 Hz. The data were stored on an SD card, with each sensor’s measurements saved in a separate file containing four columns: a timestamp and the three corresponding sensor axes.

All methods were carried out in accordance with the relevant institutional, national, and European Union guidelines and regulations governing research involving human participants. The study cohort comprised six healthy adult university students, all of whom provided written informed consent prior to participation.

The study did not involve medical or clinical experimentation, the administration of alcohol or any psychoactive substance, or the collection of biological samples. The experimental procedures consisted solely of supervised walking tasks performed with and without commercially available alcohol-impairment simulation goggles, under safe and controlled conditions.

According to the applicable national and European Union regulations, this non-interventional, minimal-risk study did not constitute a medical or clinical experiment and therefore did not require approval from an Institutional Review Board or bioethics committee. Nevertheless, the experimental protocol was reviewed internally to ensure participant safety and ethical compliance, and adhered to the principles of the Declaration of Helsinki.

All data were anonymised prior to analysis. Participation was voluntary, participants were free to withdraw at any time without penalty, and supervision was provided to mitigate foreseeable risks associated with altered visual perception.

**Figure 2:**
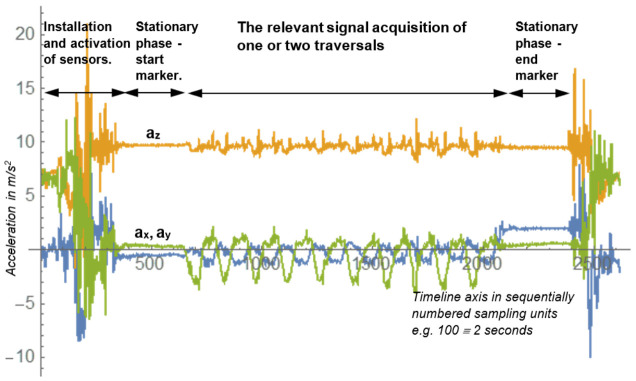
The graph presents data collected from accelerometer sensors (*a*_x_, *a*_y_, *a*_z_ in m/s^2^ units), recorded by a smartphone placed in the right trousers pocket (indicated by the red box in Figure 1) during the execution of ten steps along a straight line, without goggles. At the beginning and end of the data streams, one can observe the phases of sensor attachment and sensor activation, followed by a stationary phase (start marker), the phase of acquisition of the primary signal, another stationary phase (stop marker), and, finally, the activities associated with stopping and removing the sensors.

Each participant completed six traversals: one without goggles and one with each type of goggle. The data collected from each traversal were split into two segments (outward and return), yielding 72 recorded walk sequences, each averaging approximately 30 s in duration. Figure 2 illustrates an example of the raw acceleration signal together with the start and stop markers used to support the extraction of the walking segment for subsequent analysis.

At the initial stage of data processing, the beginning and end of each walking trial were identified in two steps. First, the approximate trial boundaries were determined manually from the raw accelerometer signal (see the small acceleration fluctuations around samples 200 and 2200 in Figure 2). These manually selected start and end markers were defined as sample indices corresponding to the onset and termination of the walking episode, after excluding the stationary periods immediately preceding gait initiation and immediately following gait termination. For each recording, these marker values were stored in the configuration file as a pair of indices (*i*_start_raw_ and *i*_stop_raw_).

In the second step, all sensor data streams were synchronised to a common uniformly sampled time axis by interpolation. As this synchronisation and resampling procedure changes the number of samples, the manually selected markers could not be transferred directly to the resampled signals. Therefore, the corresponding timestamps (*t*_start_ and *t*_stop_) were first obtained from the original accelerometer time vector using *i*_start_raw_ and *i*_stop_raw_. These timestamps were then mapped onto the synchronised time vector by selecting the first available sample positions corresponding to the same temporal boundaries. The resulting interval was subsequently used to crop all synchronised sensor channels consistently.

Finally, the manually delimited interval was automatically refined to retain only steady-state walking for further analysis. A sliding window was moved along the synchronised acceleration signal, and, for each window, an autocorrelation-based periodicity measure was calculated from the magnitude of the measured acceleration. Local maxima of this periodicity measure were interpreted as candidate locations of repeated gait patterns. The final interval used for the machine-learning analysis was defined between the earliest valid detected gait-pattern location and the latest valid detected gait-pattern location, while excluding the first and last few detected patterns. In practice, this procedure removes the gait initiation and termination phases, thereby ensuring that the retained segment corresponds to steady locomotion instead of to the transient phases at the beginning and end of the walking trial.

Therefore, for the subsequent analyses, only measurement values recorded during active movement were retained. In the example shown in Figure 2, this corresponded to samples 647 to 1027. The example plot presents raw acceleration data for a single traversal by one participant, along the OX, OY, and OZ axes. Furthermore, for subsequent processing, the OZ-axis acceleration values were offset by subtracting 9.81 m/s^2^ to provide a simplified gravity compensation. This approximation does not account for arbitrary device orientation and therefore does not provide full inertial frame normalisation. Instead, it serves as a pragmatic pre-processing choice consistent with prior smartphone-based gait studies that prioritise relative signal dynamics over absolute biomechanical, as indicated by the orange trace. The implications of this simplification are discussed in the limitations section. The acceleration components a_x_ and a_y_ shown in the graph do not exhibit zero values during periods of rest, owing to the absence of rigid fixation of the smartphone within the trousers pocket relative to the right leg. By contrast, the sensor mounted on the arm is firmly secured, thereby preventing any extraneous movement or displacement relative to the left arm. The precise estimation of a smartphone’s position and orientation has been examined in several studies [33,34]. The reliability and applicability of smartphone accelerometers for gait analysis were examined in ref. [35]. In the present investigation, this aspect is disregarded on the premise that the principal parameter of interest is the detection of gait modifications resulting from simulated alcohol-impaired vision induced using goggles.

The indoor trial was designed as a preliminary exploratory stage and should not be interpreted as the primary experimental dataset analysed in this study. Its main purpose was to identify limitations of the initial protocol and to determine which environmental conditions required stricter control in the subsequent main experiment. The dataset and final results used for the analyses reported in this paper were obtained from the outdoor experiment described in the next section.

During the indoor trial, an initial examination of the recorded inertial signals indicated relatively low variability between goggle conditions and between participants. Qualitative observations made during data acquisition also indicated that participants were generally able to follow a stable walking path, including when wearing the orange goggles, which represented the highest level of simulated impairment. It should be noted that lateral deviations from the intended walking trajectory were not quantified directly using spatial tracking, motion-capture systems, or smartphone GNSS/GPS positioning. Standard smartphone GNSS/GPS data were not used for this purpose because metre-level positioning errors are not sufficiently accurate to assess small lateral deviations in the present experimental context.

Accordingly, the environmental factors identified in the indoor trial should be interpreted as qualitative, observation-based considerations rather than as quantitatively measured variables. These factors included:

- the proximity of the corridor walls, which may have offered salient visual reference cues;
- the tiled floor surface, where clearly visible grout lines were approximately aligned with the walking direction;
- the availability of these spatial reference cues, which may have assisted spatial orientation and postural stability and, consequently, reduced the observable variability of the indoor inertial recordings.

This study comprised 96 walking trials, each conducted along a path approximately 100 m long and 2.4 m wide.

## 4. Redesign of the Experimental Setup

**Figure 3:**
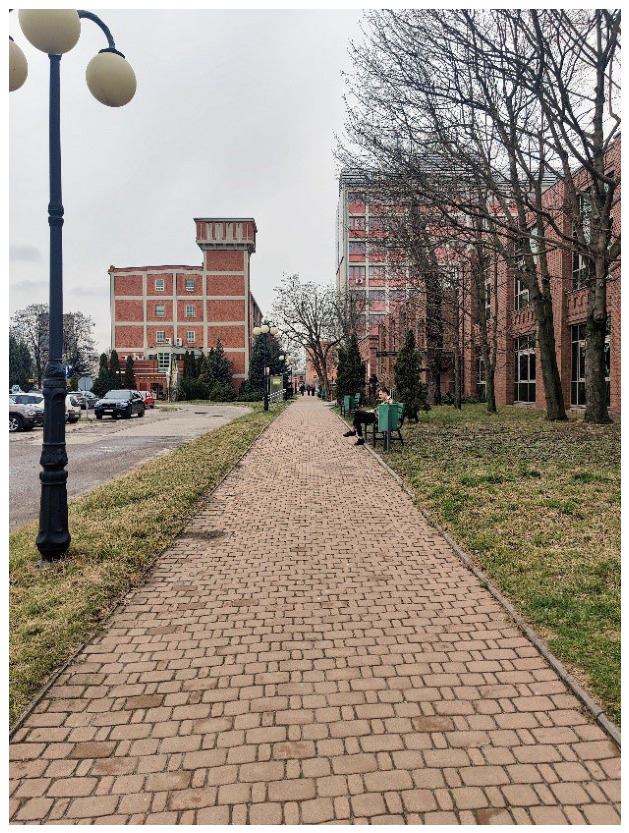
The photograph depicts a 100 m route along the path, traversed sequentially by 16 participants both with and without goggles on Lodz University of Lodz campus. The central participant is either wearing goggles or walking without them, and is equipped with two smartphones monitoring their movement, as illustrated in Figure 1. The individuals positioned on either side provide support and ensure the safety of the participant wearing the goggles.

Consequently, a second experiment was designed to eliminate these environmental aids. In this revised setup, which provided the dataset used in all subsequent analyses and for training the neural network models, participants walked 100 m in a straight line along a 2.4 m wide city pavement in an open space. The paving stones were irregularly aligned, with no straight grout lines parallel to the walking direction. Furthermore, within a 10 m radius, no fixed structures were present that could act as reference points to aid balance. Figure 3 illustrates the experimental route and the participating subjects. In this experiment, 16 participants each traversed the route six times: once in each of the six conditions (‘none’, ‘green’, ‘blue’, ‘black’, ‘red’, ‘orange’). The experimental conditions were administered in a largely fixed order, starting with the no-goggles condition and proceeding toward increasing levels of simulated impairment. No formal randomisation or counterbalancing of condition order was applied. This design choice may introduce order effects, such as task adaptation, learning of the walking route, or cumulative fatigue, which could influence the recorded gait patterns and partially confound impairment-related effects. The chosen ordering was primarily motivated by safety considerations and by the exploratory nature of the present study.

As in the initial experiment, smartphones recorded three-dimensional accelerometer and gyroscope data (six components in total from one smartphone, sampled at 50 Hz). Data were collected between 10:00 a.m. and 1:00 p.m. on a level cobblestone surface. Participants walked independently at a natural pace, while two assistants followed closely behind to ensure safety. The assistants were positioned so as to remain outside the participants’ field of view and therefore did not influence their performance. The smartphone was placed either in the right trousers pocket or on the outer side of the wrist. When worn on the wrist, the device was secured firmly, while the trousers pocket was sufficiently tight to ensure repeatability of the experiment. The initial orientation of the linear acceleration sensor axes was identical across all experiments and is illustrated in Figure 2. In most trials, the order of conditions was as follows: no-goggles, green goggles, blue goggles, black goggles, red goggles, and finally orange goggles.

Following data collection, preliminary pre-processing removed signal segments corresponding to application start-up/shutdown (using start and stop markers) and the moments when the device was inserted into or removed from the pocket or affixed to the wrist.

The recording duration was not fixed a priori and varied across trials; however, no systematic relationship between recording length and the simulated BAC level was observed. Differences in sequence length primarily reflect individual walking speed, brief pauses during the task, and natural variability in turning and stopping behaviour instead of the goggle condition itself. All reported models were trained and evaluated using only tri-axial accelerometer signals (*a*_x_, *a*_y_, *a*_z_) recorded at the leg-mounted smartphone.

Gyroscope signals and data from the wrist-mounted device were not included in the modelling pipeline described in this study.

**Figure 4:**
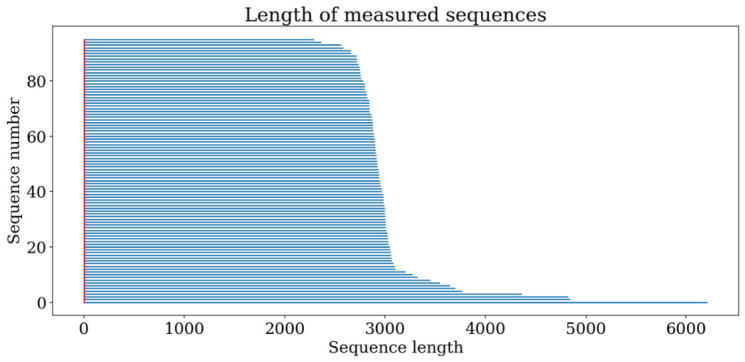
The distribution of measurement data lengths was derived from all 96 traversals performed by 16 participants in the second experiment, following post-processing and data cleaning. The data were subsequently ordered by length to facilitate clearer visualisation.

The final dataset comprised 290,919 samples recorded at a frequency of 50 Hz, corresponding to approximately 97 min of selected, usable data for subsequent analysis, distributed across 96 traversals. Consistent with previous studies, only measurements obtained during active movement were retained. Sequence lengths varied from 2293 to 6211 samples, with a mean length of 3030 samples (see Figure 4). The synthetic parameters of the processed and cleaned dataset are presented in Table 3, which summarises its overall size and key characteristics. The fully cleaned and pre-processed dataset, encompassing all volunteer traversals, is provided in the Supplementary Materials accompanying this paper and may be used free of charge, provided that a full citation of this work is given. This dataset will also be employed in subsequent analyses and considerations. A summary of the final dataset is provided in Table 4.

Longer recordings in some trials are attributable to slower walking pace and prolonged turning or stopping phases in individual participants and are not consistently associated with higher simulated BAC levels.

The considered task is formulated as a supervised multi-class classification problem. Each input sequence of accelerometer samples is assigned to one of six classes corresponding to the experimental walking conditions: walking without goggles (no simulated impairment) or walking while wearing one of five alcohol-impairment simulation goggles representing increasing levels of simulated BAC. The class labels therefore reflect levels of simulated visual impairment instead of real alcohol intoxication.

The dataset is approximately class-balanced at the traversal level, as each participant performed one traversal per goggle condition. After windowing and data augmentation, minor differences in the number of samples per class arise due to variability in sequence lengths; however, no severe class imbalance was observed.

The dataset for each traversal was partitioned so that the initial 80% was allocated to training and the remaining 20% to testing. This split was performed within each traversal, implying that training and test data originated from the same participant and walking session. Consequently, the evaluation primarily reflects within-subject performance instead of subject-independent generalisation. While this approach enables analysis of temporal consistency and model capacity, it may overestimate performance relative to subject-wise validation strategies. This limitation is explicitly acknowledged in the Discussion section. The training and test data were separated and did not overlap. Because of the very small number of participants, using a patient-level split would have resulted in inadequately small training and test sets, thereby precluding stable model training and reliable statistical evaluation. For this reason, a record-level split was used in this study, with the recognition that it may overestimate inter-subject generalisation, but it allows workable models to be developed under severe data limitations.

For data augmentation, sub-sequences were generated using a fixed-length sliding window of 256 time steps (256/50 Hz = 5.12 s) with a stride of 4, ensuring that no overlap occurred between the training and testing sets. From each original subsequence, one instance was preserved, and an additional 10 augmented sub-sequences were produced by adding white noise with a mean of 0 and a variance of either 0.1 or 0.2. The number of generated sub-sequences, together with the variance values applied for each neural network architecture, is summarised in Section 7.

## 5. Association of Simulated Alcohol-Impaired Vision with Actual BAC Values

In Ref. [36], a study involving 22 participants demonstrated that both FVGs (corresponding to BAC levels from 0.070% to 0.1% and above) and moderate alcohol intoxication (mean BAC ≈ 0.060%) produced statistically comparable impairments in complex driving tasks. For lateral control, measured via the standard deviation of lane position (SDLP), both FVGs (ES = 0.69, see Ref. [36]) and alcohol (ES = 0.65, see Ref. [36]) significantly increased SDLP from baseline, with no statistically significant difference between them. Reductions in distance headway were also moderate and similar for FVGs (ES = 0.52) and alcohol (ES = 0.40). Overlapping confidence intervals and non-significant *t*-test results support the conclusion that FVGs can replicate moderate alcohol impairment for the measured metrics. Further investigation is required to determine whether FVG-induced impairment remains comparable at higher intoxication levels.

## 6. Design of the Neural Network Architectures

The architectural components employed in this study were selected to reflect the multi-scale temporal structure of gait signals. Convolutional layers were used to capture local, short-term temporal patterns corresponding to step-level dynamics, while bidirectional LSTM layers model longer-range dependencies related to stride regularity and inter-step variability. Self-attention mechanisms were introduced to enable adaptive weighting of temporally distant yet informative signal segments, which is particularly relevant in gait data affected by transient instability induced by visual perturbation. In this study, the performance of several simple neural network architectures—comprising Convolutional Layers (Conv1D), Dropout Layers (DO), Bidirectional Long Short-Term Memory (BiLSTM), Self-Attention Layers (SelfAtt), and Fully Connected layers (FC)—was assessed (see Section 7) for gait classification based exclusively on signals from the accelerometer sensors mounted on the right leg. The networks were configured using the hyperparameters listed below, which, at this stage of the investigation, were selected on an intuitive basis. In a subsequent section of this paper, where the optimal neural network architecture is determined, these hyperparameters are discussed in detail in the context of the collected experimental data. The assumed neural network hyperparameters and their corresponding short descriptions are as follows:

- Signal Sampling Frequency—50 Hz.
- Sequence Length—The length of the input data sequences processed by the gated RNN layers. In this study, sequence lengths of 128 and 256 samples were employed to balance training time against temporal resolution.
- Hidden Layer Size—The number of units in each hidden layer of the gated RNN network, selected in the range of 64–128 to ensure adequate modelling capacity without incurring excessive computational costs.
- Number of Convolutional Layers (Conv1D)—The initial pre-processing stage, comprising two or three convolutional layers, was used to filter and detect characteristic temporal patterns in the accelerometer data.
- Filter Length—The kernel size in the convolutional layers (Conv1D), typically set within the range of 3–7 (five in this study), optimised for capturing local temporal features in EEG/ECG-type signals.
- Number of Filters—The number of feature maps in the convolutional layers, chosen in the range of 32–64 (64 in this study) to enhance feature extraction while mitigating overfitting risk.
- Self-Attention Layers: number of heads and key dimension—The number of attention heads in the Self-Attention layer was set to four, enabling the model to learn multiple feature subspaces in parallel. The dimensionality of the key vectors in the Self-Attention layer was set to 32, providing sufficient representational capacity for biomedical signal processing tasks.
- Dropout Layer Coefficient—The probability of randomly dropping units during training to prevent overfitting, set to *p* = 0.2 in this study.
- We assumed that the neural network would also account for changes in the OX/OY/OZ axis orientation during limb movement.

All models were implemented in Python using PyTorch (Version 2.7.0) together with torchvision (Version 0.22.0) and torchaudio (Version 2.7.0). Additional data processing and analysis were conducted using NumPy (Version 2.4.0), SciPy (Version 1.16.3), Pandas (Version 2.3.3), and scikit-learn (Version 1.8.0). The development environment included Spyder (Version 6.1.2) and spyder-kernels (Version 3.1.x). Experiment tracking and model evaluation were supported by MLflow (Version 2.20.2). The experiments were conducted on a workstation equipped with an NVIDIA RTX 2000 Ada Generation Laptop GPU, enabling GPU-accelerated training using CUDA 12.8.

A summary of the considered neural network architectures, along with their parameter counts and overall performance metrics, is provided in Table 5.

Based on the simulation results the best performance within the considered class of neural network architectures and the employed dataset was achieved by network A5. This architecture demonstrated superior recognition of both long- and short-term spatiotemporal patterns, leading to the highest Accuracy and Weighted F1-score values. In this network, the use of a double convolutional layer enabled the extraction of both simple and more complex temporal patterns from the analysed signal during the initial pre-processing stage. These layers also effectively reduced sensor noise and captured local features in the processed signal through the correlation of sequential data.

The incorporation of Self-Attention Layers allowed for dynamic weighting of different time steps, thereby facilitating the detection of significant and temporally distant sequences, thus extending the capabilities of both the convolutional and LSTM layers. By introducing a Layer Normalisation (LN) stage, the issues of vanishing and exploding gradients relevant in the BiLSTM-Self-Attention combination were mitigated. This layer also typically ensures consistent activation scaling, enabling higher learning rates and reducing training time. Applying a Dropout Layer (*p* = 0.2) after the sequential layer reduced the risk of overfitting, which is especially important given the limited number of samples. Finally, the Fully Connected Layer at the output stage mapped the reduced, highly selective features to the class space (six classes), minimising unnecessary complexity in the final step. We employed a self-supervised learning strategy for subsequent groups of neural network layers, specifically the Conv1D block, utilising the masked prediction model algorithm [37,38,39,40] for preliminary pretraining of the first layers of the neural network (Conv1D). At this stage, the trainable Conv1D layer was pretrained within an autoencoder framework, serving as the encoder, while the decoder was implemented as a mirrored inverse layer. This pretraining phase was conducted for three epochs, during which the network exhibited rapid convergence (with an empirically estimated exponential loss decay rate of approximately 1.29 per epoch) and learned the dominant structural patterns present in the input data. Subsequently, the entire network was trained for up to 20 epochs using the Adam optimiser with a learning rate of 1.5·10^−3^ and a batch size of 32. Cross-entropy loss was used as the optimisation criterion, and gradient clipping was applied to improve training stability. The measurement data were augmented by adding 10 additional training samples to the dataset for each original observation, with white noise applied at a variance of 0.2. In Section 7, this procedure is denoted as “Noise var. 0.2 1 → 1 + 10”. In the case of architecture A5, a lower noise variance (0.1) and a smaller number of augmented samples (5) yielded more favourable performance metrics.

## 7. Discussion

It is also evident that network A1, due to its excessive simplicity, could not classify the data at a satisfactory level. Although it maintained a reasonably balanced recognition performance across individual classes (average FPR = 3.71%), it exhibited substantial class leakage (green was reclassified into the blue category with an occurrence rate of 19%). A further limitation of this model was its high false-negative rate, which reached 1 − (min recall) = 24%. The remaining networks, A2, A3, and A5, demonstrated performance levels compatible with exploratory, low-stakes use cases in controlled experimental settings. However, in the case of A5, the use of a serial SelfAtt × 2 configuration substantially increased the number of trainable parameters. It should be emphasised that all reported performance metrics were obtained using a within-traversal 80/20 split. Thus, training and test samples were derived from the same participant and session, and the results do not represent subject-wise or cross-subject generalisation. It was observed that, across the individual folds, the results varied with a standard deviation of approximately 1.64%. Cross-validation results for the A5 model, including fold-wise performance metrics and their variability, are presented in Table 6.

A detailed class-wise breakdown of the classification performance, including precision, recall, specificity, and F1-score, is presented in Table 7. A class-wise analysis of the results indicates that walking style differs across strap colours corresponding to simulated BAC levels. Aggregated performance indicators, including minimum recall, average F1-score, class balance, and average FPR, are summarised in Table 8. In particular, lower impairment levels (green and blue straps) exhibit similar performance patterns and higher mutual confusion, suggesting subtle and closely related gait adaptations. In contrast, higher simulated levels (red and orange straps) show reduced confusion with the no-goggles condition but increased variability within their own classes, as reflected by lower recall in some models. Overall, the results suggest a gradual transition in gait behaviour with increasing simulated impairment instead of sharply separated walking styles.

Several limitations of the present study must be acknowledged. First, the experimental manipulation relied on goggle-induced visual distortion instead of actual alcohol consumption. Consequently, the observed gait alterations should be interpreted as adaptations to visual impairment, cautious behaviour, or altered sensory integration, instead of direct manifestations of alcohol-induced motor impairment. Second, the relatively small number of participants (*n* = 16) and the within-subject train–test split limit the ability to draw conclusions regarding generalisation to unseen individuals. Third, the large number of windowed samples should not be interpreted as independent observations, as they originate from a limited number of traversals. These factors necessitate cautious interpretation of classification performance and motivate future studies employing subject-wise validation and larger, more diverse cohorts.

These issues are addressed in the second part of this publication. Ongoing data collection will continue to expand the present dataset, and the published dataset will therefore be progressively enlarged as new measurements are acquired. Further experiments also appear justified across different age groups. Additionally, future work will extend the present study by incorporating both accelerometer and gyroscope signals into unified deep learning architectures, thereby enabling a systematic comparison of single- and multi-modal inertial sensing approaches.

The results of the ablation study for the A5 model, evaluating the contribution of individual accelerometer channels, are summarised in Table 9. The ablation from three (*a*_x_, *a*_y_, *a*_z_) to two linear-acceleration channels (*a*_x_, *a*_y_) did not materially change the aggregate performance metrics, while the parameter count remained nearly unchanged. This indicates that the full three-axis configuration did not derive its performance from excessive model complexity or axis-specific overfitting. However, the increase in class leakage for the most confusable class pairs suggests that the removed axis (*a*_z_) still contributed useful discriminative information for the stabilisation of local class separation (e.g., class leakage).

The ablation of an additional input component (*a*_y_) results in a further decline in all performance metrics, indicating that this component contributes useful discriminative information and offering no indication of model overfitting. A natural next step would be to examine the use of the acceleration-vector magnitude computed from the (*a*_x_, *a*_y_, *a*_z_) components. Such an analysis would, however, require removing the gravitational component from *a*_z_, since its presence would obscure the variability of the individual axes. After excluding gravitational acceleration, the vector magnitude is unlikely to provide substantially more information than the *a*_x_ component alone. The analysis of model-capacity reduction is largely illustrated by models A4 and A5 in Table 5 and Table 8. Although model A4 exhibits slightly better primary metrics, namely accuracy and weighted F1-score. However, these differences were small and the corresponding confidence intervals overlapped with those of model A5; therefore, they should not be interpreted as evidence of statistical superiority. The remaining metrics, which are more relevant for more demanding professional applications selection, are superior for model A5.

From an application-oriented perspective, the choice of model should be guided not only by aggregate performance metrics, but also by the risk profile and operational requirements of the intended use case. Model selection should therefore be determined by the intended application. Particularly in medical contexts, models are expected to satisfy a minimum recall threshold and achieve a mean F1-score of at least 90%, while preserving a satisfactory balance across classes. In such applications, reducing the number of misdiagnoses is equally critical, as reflected by the average false positive rate (avg FPR). For screening tests, even higher minimum recall values are required, together with strong class balance. By contrast, in confirmatory testing, greater emphasis should be placed on achieving lower FPR values.

In lower-stakes applications, such as entertainment and gaming, alternative trade-offs may be acceptable. For example, a model offering faster classification due to a simpler architecture or a smaller number of parameters may be preferable, even if this entails a modest decrease in classification performance.

Accordingly, for gaming-related applications, model A2, or even A1, may prove adequate, depending on the computational resources available in the target deployment environment. Conversely, for applications subject to more stringent quality requirements, models A5 and A4 would be more appropriate. In our evaluation, model A5 was preferred because it offered a better application-oriented risk profile: higher minimum recall, improved class balance, comparable FPR, and similar aggregate performance compared with model A4. Thus, model A5 should not be interpreted as being universally superior across all metrics, but rather as the preferred model under the predefined application-oriented decision criterion. Moreover, model A5 offers greater potential for further optimisation and future development, with particular emphasis on improved class balance, reduced class leakage, and a lower FPR.

## 8. Recommendations for Future Research

The research presented in this paper represents an initial but essential step. Ongoing data collection will expand the current dataset, and the published dataset will therefore be progressively enlarged as new measurements are acquired. It also appears justified to conduct further experiments across different age groups, including whether changes in the order of goggle conditions influence the results for selected participants. We expect that this will, among other things, improve the stability of the analysis based on the Leave-One-Subject-Out (LOSO) protocol. This work should be further extended by developing a neural network model that incorporates both accelerometer and gyroscope data into unified deep learning architectures, enabling a systematic comparison of single- and multi-modal inertial sensing approaches. Various neural network architectures will need to be investigated, and their performance systematically evaluated and compared with the results presented in this study. Moreover, this paper proposes initial hyperparameter values; however, these values should ultimately be optimised systematically. In addition, it is advisable to examine a broader range of neural network architectures for different exploratory and low-stakes research applications. These issues are addressed in the second part of this publication.

## 9. Conclusions

The present work demonstrates that gait alterations associated with simulated visual impairment induced by alcohol-impairment simulation goggles can be detected and classified using smartphone inertial-sensor data and appropriately designed deep learning architectures. While controlled indoor environments may artificially reduce gait variability, open and visually complex outdoor settings yielded data that are more representative of unconstrained, real-world locomotion. The reported results reflect classification performance based solely on leg-mounted accelerometer data and should not be interpreted as multimodal or multi-device fusion outcomes.

Among the evaluated models, the best-performing gaming architectures achieved results that approached performance thresholds suitable for practical, low-stakes intoxication screening based on gait alterations associated with simulated impairment (e.g., A2/A1). Model A4 achieved the strongest aggregate performance in the principal global metrics, whereas model A5 provided a more favourable profile on selected class-wise criteria and may therefore be regarded as a promising candidate for further exploratory evaluation in low-stakes contexts where better aggregate metrics are prioritised.

A key contribution of this study is the public release of a fully processed, labelled dataset comprising synchronised accelerometer and gyroscope recordings across six experimental conditions. Although the classification results reported here were obtained using accelerometer data alone, the dataset enables future investigations combining multiple inertial modalities (including gyroscope signal). By making this dataset openly available, the study aims to support further methodological development in mobile gait analysis and the classification of gait alterations associated with controlled sensory perturbation.

At the same time, the findings should not be interpreted as evidence of direct alcohol-intoxication detection or BAC estimation, and their generalisability remains limited by the modest cohort size and the within-subject evaluation design. Future work should therefore focus on enlarging the cohort, introducing subject-wise validation, and investigating real-time mobile implementations for preventive and educational use.

## Figures and Tables

**Table 1:** Typical effects of alcohol consumption on human behaviour. The values have been estimated based on the data reported in [6] and the formula proposed by Erik M. P. Widmark [4,5].

Behaviour Associated with Intoxication Level	Alcohol Dose/BAC Level [%]
Impairment begins	0.03%
Driving skills significantly affected	0.05%
Possible criminal penalties	0.08%
Legally intoxicated	0.10%
Criminal penalties	0.16%

**Table 3:** Assignment of the goggle strap colour to the manufacturer-declared Simulated Alcohol-Impaired Vision range (BAC).

Drunk Busters Totally Wasted Goggles	
Strap Colour	Simulated BAC [%]
none	0
green	0.04–0.06
blue	0.06–0.08
black	0.08–0.15
red	0.15–0.25
orange	0.26–0.35

**Table 4:** The table presents a summary of the assembled dataset.

Parameter	Value/Description
Number of participants ^1^	16
Traversals per participant per goggles type (see Table 2)	1
Distance per traversal	100 m trail/attempt
Total traversals per participant	6 (one for each goggle type)
Total traversals	16 participants · 6 traversals = 96
Age of participants	Second-semester, second-cycle students
Sensor sampling rate	50 Hz
Sensors used	Three-axis accelerometer and three-axis gyroscope (Samsung S23/S9) = 6 channels
Total samples after pre-processing	290,919 samples · 6 channels
Time points per trial (post-processing, see Figure 4)	2293–6211 (mean: 3030)

^1^ Participants were healthy adults (*n* = 16; age range: 23 ± 1 years; 50%/50% female/male), recruited from second-cycle university programmes. No participant reported neurological, musculoskeletal, or visual disorders affecting gait.

**Table 5:** The table presents a concise description of the architecture of the neural networks under investigation, along with their respective dimensions, the number of parameters determined during the network training process, and the overall accuracy and weighted F1-score indices.

Model Name	Model Architecture	Augmentation	Training & Test Sequences	Parameters Number	Accuracy 95% CI	Weighted F1-Score 95% CI
A4	Conv1D (3 → 64) × 2 → SelfAtt → LN → BiLSTM (64 → 32 × 2) → DO → FC(32 × 2 → 6)	Noise var. 0.2 1 → 1 + 10; Sliding-Window(fixed length) with stride = 4.	[312,570; 256; 3] [8406; 256; 3]	38,470	72.42% [−5.11 pp; +4.61 pp]	72.29% [−4.63 pp; +4.77 pp]
A5	Conv1D (3 → 64) × 2 → SelfAtt × 2 → LN → BiLSTM (64 → 32 × 2) → DO → FC(32 × 2 → 6)	Noise var. 0.1 1 → 1 + 10; Sliding-Window(fixed length) with stride = 4.	[312,570; 256; 3] [8406; 256; 3]	72,006	71.36% [−2.79 pp; +2.65 pp]	71.49% [−2.68 pp; +2.79 pp]
A2	BiLSTM (3 → 32)→ DO → FC(32 × 2 → 6)	Noise var. 0.2 1 → 1 + 10; Sliding-Window(fixed length) with stride = 4.	[312,570; 256; 3] [8406; 256; 3]	9862	71.39% [−2.70 pp; +2.54 pp]	71.32% [−2.65 pp; +2.54 pp]
A3	Conv1D (3 → 64) × 3 → LN → BiLSTM (64 → 32 × 2) → DO → FC(32 × 2 → 6)	Noise var. 0.2 1 → 1 + 10; Sliding-Window(fixed length) with stride = 4.	[312,570; 256; 3] [8406; 256; 3]	50,822	70.21% [−2.79 pp; +2.66 pp]	70.15% [−2.79 pp; +2.73 pp]
A1	Conv1D (3 → 64) ×3 → DO → FC(32 × 2 → 6)	Noise var. 0.2 1 → 1 + 10; Sliding-Window(fixed length) with stride = 4.	[312,570; 256; 3] [8406; 256; 3]	1030	65.41% [−2.67 pp; +2.5 pp]	64.81% [−2.62 pp; +2.57 pp]

**Table 6:** The Cross-Validation results for the A5 model. The aggregated indicators synthetically present the recognition within each of the six classes. The proportion of cases in which the system incorrectly classifies an event as positive, although in reality it is negative (Type I error, FPR), is calculated using the following formula: (FPR for class A) = 1 − (Specificity of super-class A). Avg and std denote the mean and standard deviation, respectively. All values are rounded to two decimal places, whereas the leakage values are rounded to the nearest whole number. The lower and upper bounds of the 95% confidence interval for the reported metrics were estimated from the fold-wise results using Student’s t-distribution and are reported in percentage points (pp) relative to the corresponding point estimate and the respective metric.

Model/ Kfold	Accuracy	Weighted F1-Score	Min Recall	Avg F1-Score	Class Balance	Avg FPR	Class Leakage/ the Worst Mistake
A5/1	71.21%	71.25%	63.49%	71.17%	14.27 pp	5.76%	blue → green (24%) red → black (21%)
A5/2	75.01%	75.02%	68.17%	74.93%	13.98 pp	5.00%	blue → green (20%) red → black (16%)
A5/3	75.08%	75.13%	67.49%	75.04%	16.73 pp	4.98%	blue → green (17%) red → black (15%)
A5/4	74.86%	74.89%	65.43%	74.80%	18.77 pp	5.03%	red → black (16%) blue → green (15%)
A5/5	73.76%	73.81%	64.24%	73.72%	18.00 pp	5.24%	red → black (16%) green → blue (16%)
avg	73.98%	74.02%	65.76%	73.93%	16.35 pp	5.20%	
std	±1.64%	±1.64%	±2.02%	±1.63%	±2.16 pp	±0.33%	
95%CI	±2.04 pp	±2.04 pp	±2.51 pp	±2.02 pp	±2.68 pp	±0.41 pp	

**Table 7:** Values of the fundamental metrics describing the classification quality for each class. For the reported results, 95% Wilson confidence intervals (CIs) were estimated for the accuracy metric [41], while 95% bootstrap CIs were estimated for Recall, F1-score, class balance, and FPR [42]. The intervals are expressed in percentage points (pp) relative to the corresponding point estimate. The values presented in square brackets indicate the mean lower and upper deviations of the estimated CIs, along with their standard deviations, computed across the six analysed classes and expressed in pp with respect to the corresponding metric. Thus, for example, 87.09% with [−11.91 (±1.77) pp, +7.81 (±3.59) pp] corresponds to the absolute interval [75.18% ± 1.77 pp, 94.90% ± 3.59 pp], where the uncertainties refer to the corresponding interval limits.

Model	Metric 95% CI	Recognised Label					
		None	Green	Blue	Black	Red	Orange
A4	Precision [−11.91 (±1.77) pp, +7.81 (±3.59) pp]	87.09%	90.96%	67.61%	79.04%	57.69%	61.73%
	Recall [−12.04 (±2.59) pp, +8.43 (±3.81) pp]	71.97%	65.14%	86.91%	75.05%	48.24%	86.27%
	Specificity [−2.94 (±0.79) pp,+1.67 (±0.89) pp]	97.81%	98.73%	91.42%	95.65%	93.31%	90.05%
	F1-score [−9.22 (±1.99) pp, +7.79 (±2.00) pp]	78.81%	75.91%	76.05%	76.99%	52.55%	71.96%
A5	Precision [−6.86 (±0.30) pp, +5.91 (±0.43) pp]	82.18%	67.11%	68.98%	81.56%	63.38%	64.91%
	Recall [−6.98 (±0.34) pp, +6.20 (±0.60) pp]	82.98%	69.21%	69.36%	73.66%	65.00%	67.22%
	Specificity [−1.70 (±0.38) pp, +1.33 (±0.41) pp]	96.30%	93.37%	93.57%	96.36%	92.90%	93.24%
	F1-score [−5.75 (±0.29) pp, +5.16 (±0.40) pp]	82.58%	68.15%	69.17%	77.41%	64.18%	66.05%
A2	Precision [−6.67 (±0.43) pp,+5.60 (±0.96) pp]	79.15%	82.60%	63.63%	87.07%	59.64%	63.82%
	Recall [−6.87 (±0.65) pp,+6.07 (±1.02) pp]	72.52%	72.56%	87.47%	64.76%	51.83%	78.83%
	Specificity [−1.65 (±0.49) pp,+1.28 (±0.52) pp]	96.07%	97.01%	89.70%	97.90%	93.37%	91.69%
	F1-score [−5.50 (±0.61) pp,+5.05 (±0.46) pp]	75.69%	77.26%	73.67%	74.28%	55.46%	70.54%
A3	Precision [−7.12 (±1.08) pp, +6.41 (±1.13) pp]	88.01%	72.64%	62.36%	76.01%	58.89%	66.29%
	Recall [−6.88 (±0.41) pp, +6.27 (±0.85) pp]	72.40%	73.75%	77.57%	73.30%	48.73%	74.70%
	Specificity [−1.79 (±0.31) pp, +1.43 (±0.34) pp]	97.99%	94.54%	90.43%	95.06%	93.47%	92.80%
	F1-score [−5.75 (±0.83) pp, +5.42 (±0.94) pp]	79.45%	73.19%	69.14%	74.63%	53.33%	70.25%
A1	Precision [−6.75 (±0.60) pp, +5.77 (±0.88) pp]	68.03%	78.96%	65.54%	69.33%	57.74%	51.61%
	Recall [−6.79 (±0.49) pp,+5.90 (±0.86) pp]	68.55%	73.73%	72.70%	77.84%	35.15%	61.84%
	Specificity [−1.65 (±0.32) pp, +1.28 (±0.34) pp]	93.37%	96.16%	92.12%	92.48%	95.13%	89.22%
	F1-score [−5.32 (±0.61) pp, +5.00 (±0.62) pp]	68.29%	76.25%	68.93%	73.34%	43.70%	56.27%

**Table 8:** The aggregated indicators synthetically present the recognition within each of the six classes. The proportion of cases in which the system incorrectly classifies an event as positive, although in reality it is negative (Type I error, FPR), is calculated using the following formula: (FPR for class A) = 1 − (Specificity of super-class A). All values are rounded to two decimal places, whereas the leakage values are rounded to the nearest whole number. For the reported results, 95% Wilson confidence intervals (CIs) were estimated for the accuracy metric [41], while 95% bootstrap CIs were estimated for Recall, F1-score, class balance, and FPR [42]. The intervals are expressed in percentage points (pp) relative to the corresponding point estimate. The values presented in square brackets indicate the mean lower and upper deviations of the estimated CIs, expressed in pp with respect to the corresponding metric.

Model	Min Recall 95% CI	Avg F1-Score 95% CI	Class Balance 95% CI	Avg FPR 95% CI	Class Leakage/ the Worst Mistake
A4	48.24% [−13.93 pp,+10.83 pp]	72.05% [−4.35 pp,+4.01 pp]	38.67 pp [−11.48 pp,+14.57 pp]	5.50% [−0.81 pp,+0.87 pp]	green → blue (27%) red → orange (24%) red → black (20%)
A5	65.00% [−7.16 pp,+3.58 pp]	71.25% [−2.92 pp,+2.66 pp]	17.99 pp [−4.64 pp,+10.19 pp]	5.71% [−0.55 pp,+0.57 pp]	green → blue (23%) orange → red (18%)
A2	51.83% [−7.65 pp,+3.38 pp]	71.15% [−2.71 pp,+2.53 pp]	35.63 pp [−5.32 pp,+9.62 pp]	5.71% [−0.52 pp,+0.54 pp]	red → orange (27%) green → blue (24%) black → red (23%)
A3	48.73% [−2.86 pp,+2.75 pp]	70.00% [−2.84 pp,+3.03 pp]	28.84 pp [−9.09 pp,+8.72 pp];	5.95% [−0.60 pp,+0.55 pp]	green → blue (21%) red → orange (21%)
A1	35.15% [−7.25 pp,+6.90 pp];	64.46% [−2.61 pp,+2.63 pp]	42.68 pp [−8.33 pp,+8.84 pp]	6.92% [−0.55 pp,+0.52 pp]	red → orange (28%) red → black (21%) orange → none (17%)

**Table 9:** The table reports the performance metrics obtained for the A5 model after the ablation of selected input components. The aggregated indicators summarise the model’s classification performance across the six classes in a concise form. The proportion of cases in which the system incorrectly classifies an event as positive, although in reality it is negative (Type I error, FPR), is calculated using the following formula: (FPR for class A) = 1 − (Specificity of super-class A). Avg and std denote the mean and standard deviation, respectively. All values are rounded to two decimal places, whereas the leakage values are rounded to the nearest whole number. For the reported results, 95% Wilson confidence intervals (CIs) were estimated for the accuracy metric [41], while 95% bootstrap CIs were estimated for Recall, F1-score, class balance, and FPR [42]. The intervals are expressed in percentage points (pp) relative to the corresponding point estimate.

Model/ Channels	Parameters Number	Accuracy 95% CI	Weighted F1-Score 95% CI	Min Recall 95% CI	Avg F1-Score 95% CI	Class Balance 95% CI	Avg FPR 95% CI	Class Leakage/ the Worst Mistake
A5/ *a*_x_, *a*_y_, *a*_z_	72,006	71.40% [−2.79 pp; +2.65 pp]	71.48% [−2.68 pp; +2.79 pp]	65.00% [−7.4 pp; +5.0 pp]	71.25% [−2.65 pp; +2.73 pp]	17.99 pp [−5.55 pp; +9.90 pp]	5.71% [−0.56 pp; +0.54 pp]	green → blue (23%) orange → red (18%)
A5/ *a*_x_, *a*_y_	71,814	71.36% [−2.85 pp; +2.74 pp]	71.49% [−2.88 pp; +2.92 pp]	65.17% [−7.47 pp; +7.16 pp]	71.28% [−2.81 pp; +2.72 pp]	12.53 pp [−8.51 pp; +9.03 pp]	5.72% [−0.58 pp; +0.57 pp]	green → blue (24%) orange → red (20%)
A5/ *a*_x_, *a*_z_	71,814	68.50% [−2.91 pp; +2.82 pp]	68.79% [−2.81 pp; +2.77 pp]	57.53% [−7.33 pp; +5.89 pp]	68.70% [−2.89 pp; +2.64 pp]	21.02 pp [−8.81 pp; +9.87 pp]	6.29% [−0.55 pp; +0.58 pp]	black → red (25%) green → blue (24%) red → orange (17%) orange → red (17%)
A5/ *a*_x_	71,622	62.62% [−2.91 pp; +2.83 pp]	62.80% [−2.95 pp; +2.86 pp]	54.08% [−7.30 pp; +3.28 pp]	62.65% [−2.98 pp; +2.70 pp]	14.66 pp [−5.78 pp; +9.43 pp]	7.47% [−0.57 pp; +0.58 pp]	green → blue (25%) red → black (20%) orange → red (18%)
A5/ *a*_z_	71,622	64.38% [−2.96 pp; +2.95 pp]	64.43% [−2.99 pp; +2.94 pp]	57.67% [−6.42 pp; +6.39 pp]	64.25% [−2.92 pp; +2.72 pp]	12.84 pp [−6.92 pp; +9.15 pp]	7.12% [−0.58 pp; +0.58 pp]	blue → green (19%) red → black (19%) red → orange (18%) green → blue (17%)

## Data Availability

The dataset generated and analysed in this study is not publicly deposited but is available from the corresponding author upon reasonable request. All analyses presented in this manuscript were conducted using this internally collected dataset. The released dataset includes accelerometer and gyroscope recordings from both device locations. These additional signals are provided to facilitate future multimodal and multi-sensor analyses but are outside the scope of the present work.

## Supplementary Materials

The following supporting information can be downloaded at: https://www.mdpi.com/article/10.3390/s26103038/s1.
